# Dacryoadenitis: do not forget ANCA vasculitis

**DOI:** 10.22336/rjo.2024.35

**Published:** 2024

**Authors:** Abir Derbel, Raida Ben Salah, Rihem Boukhzar, Faten Frikha, Sameh Marzouk, Zouhir Bahloul

**Affiliations:** * Department of Internal Medicine, Hedi Chaker Hospital, Sfax, Tunisia

**Keywords:** dacryoadenitis, ANCA vasculitis, pachymeningitis

## Abstract

**Objective:** This paper aimed to describe another form of aggressive limited Granulomatosis with polyangiitis (GPA) revealed by dacryoadenitis.

**Methods and results:** We report an unusually limited GPA in a 48-year-old man presenting with bilateral proptosis. She had never presented kidney or pulmonary manifestations, but her disease was persistently active including oto-rhino-laryngological manifestations, dacryoadenitis, and neurological manifestations unresponsive to corticosteroids and immunosuppressors.

**Discussion:** Granulomatosis with polyangiitis (GPA) is an auto-immune inflammatory vasculitis. Involvement of lacrimal glands as the first presentation is uncommon. It is characterized by the development of granulomas. Patients with orbital mass without lacrimal gland involvement have a higher rate of systemic disease, a severe clinical course, and a higher rate of recurrences. A patient with dacryoadenitis seems to be with a good prognosis. Eye manifestations were significantly more common in patients with pachymeningitis. MPO-ANCA-positive pachymeningitis was more frequent in older female patients. PR3-ANCA-positive pachymeningitis had more severe neurological damage. Induction treatment consists of intravenous methylprednisolone (IV) associated with cyclophosphamide.

**Conclusion:** Faced with dacryoadenitis, it is important to screen for ANCA-associated vasculitis.

**Abbreviations:** GPA = Granulomatosis with polyangiitis, ANCA = Antineutrophil Cytoplasmic Antibodies

## Introduction

Granulomatosis with polyangiitis (GPA), formerly known as Wegener’s disease is an anti-neutrophil cytoplasm antibody (ANCA)-associated small vessel vasculitis (AAV) characterized typically by a triad of symptoms combining oto-rhino-laryngology, renal and pulmonary manifestations. This systemic inflammatory disease had a wider spectrum of clinical symptoms depending on the organ involvement [**1**]. It can affect the heart, eyes, skin, joints, and nervous system. GPA was classified into generalized and localized systemic phenotypes [**1**,**2**]. The systemic form was characterized by pauci-immune focal necrotizing glomerulonephritis and necrotizing granulomatosis inflammation of the respiratory tract, but the disease is not limited to pulmonary or renal systems [**2**]. The inflammation can involve whole body organs. In localized form, lesions were limited in some organs: the nasopharynx, throat, middle ear, and orbit. Lacrimal gland involvement can occasionally be the sole presentation preceding any other organ manifestation or systemic disease [**3**]. Localized form affecting the meninges is also uncommon [**4**].

In this report, we describe an unhabitual presentation of GPA revealed by dacryoadenitis, a patient who presented with frontal pachymeningitis during the disease.

## Case report

In February 2015, a 48-year-old male was referred to our Department of Internal Medicine for bilateral proptosis.

**Fig. 1:**
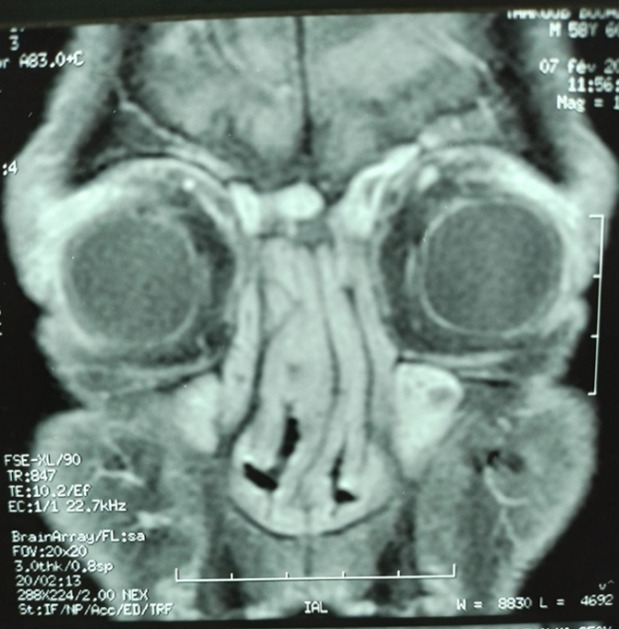
Coronal MRI (T1 sequence) revealing dacryoadenitis

During anamnesis, we found bilateral nasal obstruction, epistaxis, otorrhea, and intensive headache. On physical examination, we objectified bilateral proptosis. The blood pressure was at 120/70 mm Hg. Cardiopulmonary auscultation was normal. Neurological examination did not reveal pathologic findings. Oto-rhino-laryngological examination showed chronic sinusitis. Nasal middle meatotomy and anterior ethmoidectomy were practiced without improvement. Initial laboratory workup was significant for elevated inflammatory markers (C reactive protein: 24 mg/L, erythrocyte sedimentation rate: 97 mm/1st hour). We found 12250 WBC/µL with 8000/µL neutrophils and 600/µL eosinophils on blood count. Urinalysis was normal. Chest X-rays were normal. Facial bone computed tomography (CT) showed signs of chronic sinusitis with polypoid diffuse filling of the supra-frontal maxillary sinuses and ethmoidal cells. Orbital MRI revealed bilateral lacrimal gland enlargement with homogeneous contrast enhancement polypoid thickening of maxillary, ethmoidal, and frontal sinuses. No bone erosion was observed (**Fig. 1**). 

Cavum biopsy revealed signs of vasculitis with inflammation composed of granuloma and reactive lymphoid hyperplasia of the nasopharyngeal mucosa. The immunological study showed positive p-ANCA anti-MPO. Diagnosis of granulomatosis with polyangiitis with its localized form (ENT and ocular involvement) was retained and the patient was treated with high doses of steroids, being started on calcium substitution and antibiotic-prophylaxis (Bactrim*). He developed diabetes, which was treated with insulin. 

Progress was marked by the onset of acute diplopia with vertigo and unsteadiness in walking and standing. Neurological examination showed worsening of ptosis, right eye adduction palsy, myosis, and bilateral ptosis. He walked in small steps. Brain MRI revealed signs of stroke in the third nerve core III. He was treated with Aspegic 100 mg/day and statin 40 mg/day. Progress showed good outcomes in the 5th week of treatment. He had no otorrhea, otalgia, or nasal obstruction. We observed that proptosis regressed along with the improvement of right eye adduction. Biological findings revealed improvement of inflammatory markers (CRP=1).

**Fig. 2:**
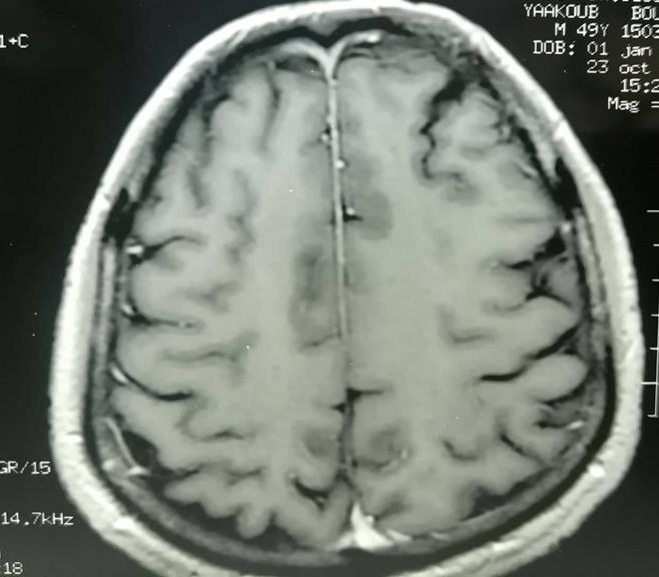
Cerebral MRI revealing frontal pachymeningitis

In October 2015, he presented with a neurological flare. He had intensive holo cranial headache associated with orbital pain and paralysis of the eye muscles. Cerebral MRI (**Fig. 2**) found frontal pachymeningitis with a granulomatosis aspect. He was treated with high doses of steroids starting with 3 pulses of methylprednisolone daily. He also received immunosuppressive therapy: cyclophosphamides at 1 g every month. At the third cure, he developed a recurrence of ocular symptoms related to bilateral retrobulbar optic neuritis. The therapeutic decision was to switch cyclophosphamide to methotrexate with high doses of steroids. Progress showed initial improvement with headache recurrence during the digression of corticosteroids at a dose of 35 mg/day. A dose of 20 mg of prednisone was maintained with good progress.

## Discussion

Granulomatosis with polyangiitis (GPA) is a multisystem auto-immune and inflammatory vasculitis that typically involves the upper airways, lungs, and kidneys. Inflammatory destructive lesions may develop in almost all organs [**1**].

Involvement of lacrimal glands as the first presentation is uncommon [**2**-**4**]. Diagnosis of orbital GPA can be difficult because of non-specific clinical features and the absence of systemic progression [**5**]. Making an early diagnosis is extremely important because the disease is locally destructive with irreversible functional loss in some cases. The occurrence of orbital involvement in GPA varies from 15 to 60% of patients [**5**-**8**]. It was associated with significant morbidity. Orbital manifestations may appear later in the follow-up, being most commonly reported with concomitant systemic manifestations [**8**]. Bilateral involvement is reported in 30 to 40% of cases [**9**]. Some authors divided orbital involvement into 4 groups that are linked to the inflammation of the structure of the eye: 1 - lesions of the orbit and appendages; 2 - those of the conjunctiva and fibrous tunic of the eyeball; 3 - those of the retina; 4 - those of the optic nerve [**6**]. 

Ocular affection is characterized by the development of granulomas in the orbit leading to dacryoadenitis, inflammatory infiltration of the surrounding tissues, including oculomotor muscles, and destruction of orbital bony walls. Nonspecific conjunctivitis and episcleritis are commonly observed. Two recent studies reported episcleritis and scleritis as the most common manifestations [**10**,**11**]. 

GPA can cause nodular, diffuse, or necrotizing scleritis. The retina is rarely involved and is described as a sporadic case in the literature. The optic nerve is mainly damaged due to orbit compression by inflamed tissue. Nevertheless, the lesion is less common due to the ischemic mechanism. In a study published in 2018 [**8**], whose aim was to distinguish different forms of orbital involvement in GPA and to compare their clinical course and outcomes, three types were proposed: orbital mass without primary lacrimal gland involvement, dacryoadenitis, and extraocular myositis. 

Patients with orbital mass without lacrimal gland involvement have a higher rate of systemic disease, a severe clinical course associated with other ophthalmic manifestations (necrotizing scleritis, orbital walls destruction), a relatively worse outcome with a high level of morbidity (optic nerve atrophy, anophthalmos, strabismus), and a higher rate of recurrences. On the other hand, a patient with dacryoadenitis seems to be not severe, with a good prognosis. 

The other particular manifestation found in our observation was the pachymeningitis. Eye manifestations were significantly more common in patients with pachymeningitis than without pachymeningitis [**12**].

The prevalence of pachymeningitis was significantly lower in newly diagnosed ANCA-associated vasculitis than in relapsed diseases. The theory of involvement of the central nervous system, which could be developed in the chronic phase of GPA, is confirmed by that [**13**]. Pachymeninges are more affected than leptomeninges [**14**].

MPO-ANCA-positive pachymeningitis was more frequent in older female patients. The lesions were rather limited to the dura mater and upper airways than generalized systemic progression. PR3-ANCA-positive pachymeningitis had more severe neurological damage and generalized disease progression [**15**]. Overall, the involvement of the central nervous system is considered an organ-threatening manifestation of GPA, notably in meningeal inflammation. Induction treatment consists of high doses of oral steroids initiated by 3 pulses of intravenous methylprednisolone (IV) associated with cyclophosphamide [**16**]. Cyclophosphamides were prescribed on intravenous pulses 1 g/cure every 2 weeks initially, then every month. The daily oral regimen can be a safe alternative [**17**,**18**]. Rituximab was considered an efficient therapy in the induction phase as seen with cyclophosphamides in two randomized trials [**19**].

## Conclusion

The diagnosis of GPA remains a big challenge and can be underdiagnosed because of atypical onset manifestations. The current report outlined a new case of GPA revealed by proptosis. Dacryoadenitis is one of many other orbital manifestations of GPA. Involvement of the eye could lead to visual loss and facial deformity. That is why, we insist on the importance of early diagnosis. In addition, the ophthalmologist’s crucial role is to think about GPA when faced with dacryoadenitis, or other orbital manifestations, even if isolated or appearing during the progress of the disease. Collaboration between specialists, including rheumatologists, internists, and ophthalmologists is often crucial to ensure optimal patient outcomes.

**Conflict of Interest Statement**

The authors state no conflict of interest.

**Informed Consent and Human and Animal Rights Statement**

Informed consent has been obtained from the individual included in this study.

**Authorization for the use of human subjects**

The research related to human use complies with all the relevant national regulations, and institutional policies, as per the tenets of the Helsinki Declaration.

**Acknowledgments**

None.

**Sources of Funding**

None.

**Disclosures**

None.
